# Ruptured Aneurysm of the Sinus of Valsalva Causing Pulmonary Embolism: A Rare Association

**DOI:** 10.1055/s-0040-1722099

**Published:** 2020-12-11

**Authors:** Francisco V. C. Barroso, Isabela T. Takakura, Ricardo C. Reis, Acrisio S. Valente, Neiberg A. Lima, Camila R. F. Gomes, José Justo Neto Júnior, Carlos R. M. Rodrigues Sobrinho

**Affiliations:** 1 Department of Cardiology and Cardiovascular Surgery, Federal University of Ceará (UFC), Walter Cantídio University Hospital, Fortaleza, Ceará, Brazil; 2 School of Medicine, University of Fortaleza (UNIFOR), Fortaleza, Ceará, Brazil; 3 Department of Pneumology, Federal University of Ceará (UFC), Walter Cantídio University Hospital, Fortaleza, Ceará, Brazil

**Keywords:** sinus of Valsalva's aneurysm, pulmonary embolism, aneurysm

## Abstract

Aneurysms of the sinus of Valsalva are rare. Unruptured sinus of Valsalva aneurysm is usually asymptomatic and rarely presents as right ventricular outflow obstruction, myocardial infarction as a result of coronary artery compression, conduction disturbances, or endocarditis. They have only been reported as the presumed source of embolism in six cases. We report a patient with right sinus of Valsalva rupture to the right atrium and embolization of aneurysm contents to the pulmonary vasculature.

## Introduction

Aneurysm of the sinus of Valsalva is a rare cardiac abnormality occurring in between 0.09 and 0.15% of cases, and accounts for up to 3.5% of all congenital cardiac anomalies. This type of aneurysm is typically congenital and may be associated with heart defects. It concerns a lack of continuity between the aortic media and aortic annulus leading to subsequent weakening, avulsion, and aneurysmal formation. It is sometimes associated with Marfan's or Loeys–Dietz syndrome but may also result from Ehlers–Danlos syndrome, atherosclerosis, syphilis, cystic medial necrosis, chest injury, or infective endocarditis. There is a male to female predominance of 4:1, with the highest incidence in Asian populations. 1 Unruptured sinus of Valsalva aneurysm is usually asymptomatic and rarely presents as right ventricular outflow obstruction, myocardial infarction as a result of coronary artery compression, conduction disturbances, or endocarditis. 2 However, a ruptured aneurysm typically leads to an aortocardiac shunt and progressively worsening heart failure. Sinus of Valsalva's aneurysms has been reported as the presumed source of embolism in only six cases. 2 3 4 5 6 7 Herein, we report a patient with the rupture of the right sinus of Valsalva into the right atrium and concurrent pulmonary thromboembolism.

## Case Presentation

**Fig. 1:**
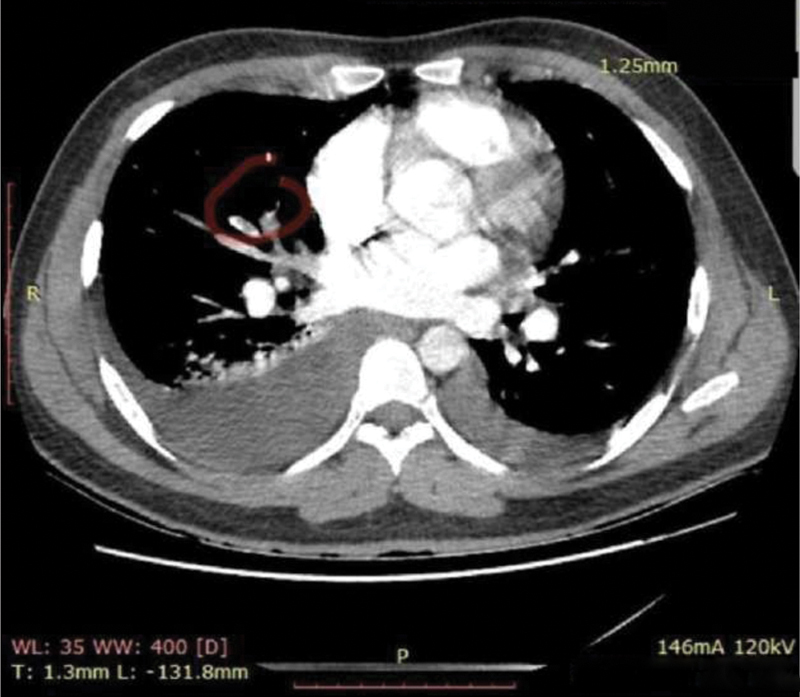
Chest computed tomography angiography demonstrating filling failure in the segmental artery of the right lower lobe (red circle).

**Fig. 2:**
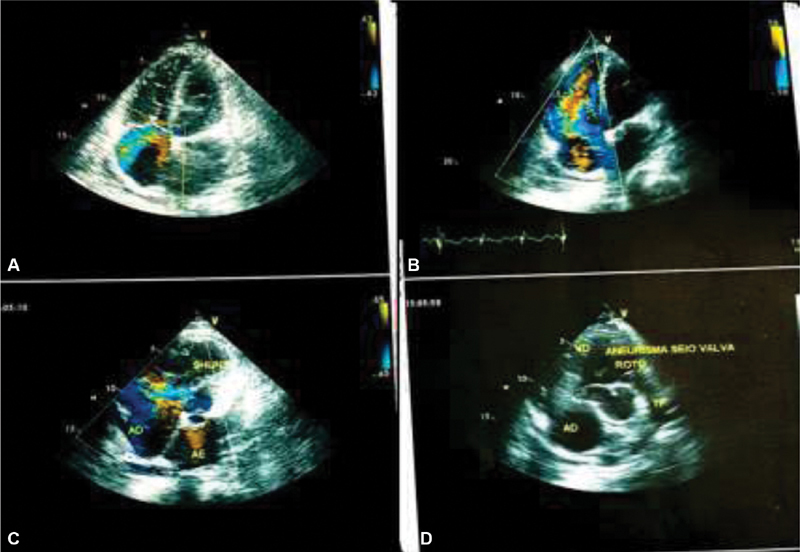
( **A-C** ) Transthoracic echocardiogram showing the aortic shunt to the right heart chambers; ( **D** ) the ruptured sinus of Valsalva aneurysm.

**Fig. 3:**
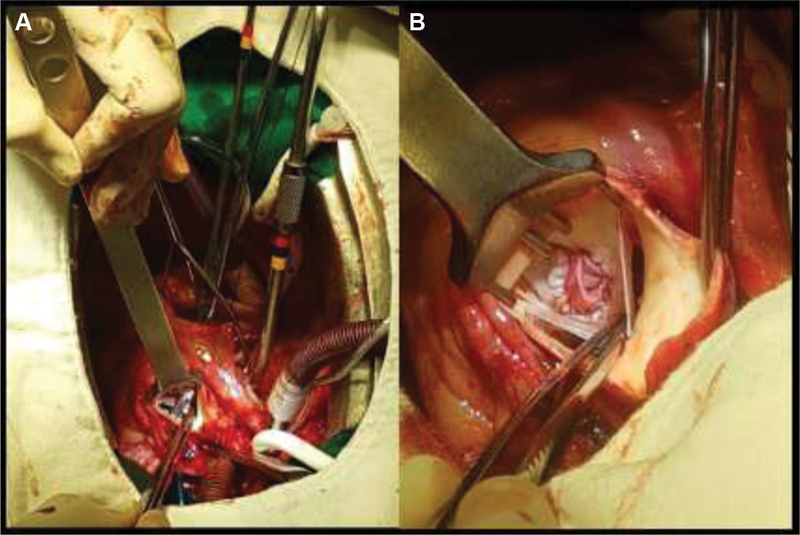
( **A** ) Fistula between the aorta and right atrium (Kelly's forceps all the way in); ( **B** ) surgical closure of the fistula between the aorta and right atrium (view of the aorta).

A 29-year-old man with no significant past medical history complaining of sudden onset dyspnea was admitted through the emergency department. His physical examination was significant for a 4 +/6+ systolic–diastolic heart murmur, elevated jugular venous pressure, and +/4+ ankle swelling. His admission electrocardiogram showed a sinus rhythm with a heart rate of 90 bpm, and serum analysis showed D-dimer and Type B Natriuretic peptide concentrations of 3,000 ng/mL and 1,700 ng/mL, respectively. He underwent a chest computed tomography angiography that showed absent opacification of the segmental artery to the right lower lobe filling ( Fig. 1 ) confirming a pulmonary embolism. Due to these abnormal findings, the patient underwent a transthoracic echocardiogram, which revealed a shunt between the aorta and right atrium ( Fig. 2 ). The lower limb venous Doppler was negative for thrombosis. Evaluation for coagulation disorders was negative. The patient denied any signs or symptoms of cardiac ischemia or other embolic complications such as stroke. During the hospital stay, he developed acute renal failure due to the difficult management of high output heart failure caused by aortic fistula. He underwent hemodialysis three times, which improved renal function. Subsequently, the patient underwent surgical repair ( Fig. 3 ) with a bovine pericardium patch using extracorporeal circulation without any complications during or after the procedure. The patient was discharged 1 week after surgery. Postoperative echocardiography showed a mild shunt from the sinus of Valsalva to the right atrium and moderate pericardial effusion without hemodynamic compromise. Valsalva sinus biopsy of the ruptured aorta revealed areas of myxoid degeneration in the vascular wall.

## Discussion

Aneurysmal dilatation of the sinus of Valsalva is a rare condition due to dysplasia of the vascular media. It is a rare cardiac anomaly that may be either acquired or congenital. 8 The congenital form of the disorder arises from the absence of continuity between the aorta and annulus fibrosus. 9 In this case, biopsy of the ruptured aorta revealed areas of myxoid degeneration in the vascular wall. Most likely, the artery wall was fragile due to the congenital anomaly in its formation. The most common anatomic location is from the right sinus of Valsalva into the right atrium.

Aneurysms of the sinus of Valsalva are usually diagnosed as incidental findings or systematically after an acute rupture into an adjacent cardiac structure. 10 There have also been cases of right ventricular outflow obstruction, conduction disturbances, and endocarditis associated with this anomaly.

Embolic events associated with unruptured sinus of Valsalva aneurysms are extremely rare. 11 A review of the literature revealed six cases of unruptured sinus of Valsalva aneurysms presenting with embolization (four cases with cerebral embolism, one with peripheral arterial emboli, 7 and one with renal infarction 6 ). We found no reports of embolism in patients with ruptured sinus of Valsalva aneurysms before surgery. Before rupture, aneurysms of the sinus of Valsalva may present with thrombus originating in the aneurysmal sac. 10 An abnormal vortex flow in the sinus of Valsalva aneurysm may contribute to thrombus formation. Since the vortex flow tends to preserve the kinetic energy of the bloodstream, the center of the vortex tends to be stagnant. This stagnation may be augmented by aneurysmal enlargement. 12 13 A case of rapid thrombus growth in the unruptured aneurysm of the sinus of Valsalva following coronary angiography has been reported. 12

In this case, our hypothesis was that pulmonary thromboembolism was due to the formation of thrombus in the aneurysmal sac of the sinus of Valsalva, which ultimately embolized to the lung after rupturing into the right atrium. No other cause for pulmonary thromboembolism was found.

The optimal treatment for ruptured sinus of Valsalva aneurysm is a surgical procedure and one of the following two main procedures may be chosen: a percutaneous closure or surgical repair. Patients with a rupture opening diameter exceeding 10 mm, combined with other cardiac lesions, or with complex anatomy should undergo surgical repair. 14 Surgical repairs should be considered for unruptured aneurysms of the sinus of Valsalva as a potential source of embolization, regardless of thrombus formation.

The present case highlights the possibility of embolization to the pulmonary vasculature from the right side of the heart when rupture of the sinus of Valsalva aneurysm occurs into the right heart chambers.
